# Effect of *Elaeagnus angustifolia* Honey in the Protection Against Ethanol-Induced Chronic Gastric Injury via Counteracting Oxidative Stress, Interfering with Inflammation and Regulating Gut Microbiota in Mice

**DOI:** 10.3390/foods14091600

**Published:** 2025-05-01

**Authors:** Min Zhu, Jiayan Yang, Haoan Zhao, Yu Qiu, Lin Yuan, Jingyang Hong, Wei Cao

**Affiliations:** 1 College of Smart Agriculture (Research Institute), Xinjiang University, Urumqi 830049, China; minna9071@126.com (M.Z.); yangjiayan_m@163.com (J.Y.); 107552303732@stu.xju.edu.cn (Y.Q.); yuanlin667788@163.com (L.Y.); 2 College of Food Science and Technology, Northwest University, Xi’an 710069, China; zhaohaoan@nwu.edu.cn; 3 Bee Product Research Center of Shaanxi Province, Xi’an 710065, China

**Keywords:** honey, gastric injury, antioxidant, inflammation, gut microbiota

## Abstract

Chronic alcohol consumption is a major contributor to gastric injury, yet current therapeutic strategies predominantly rely on chemical agents with limited efficacy and potential side effects. Natural products, with their multi-target biocompatibility and safety advantages, offer promising alternatives for gastric protection. We examined the phenolic compounds of *Elaeagnus angustifolia* honey (EAH) and investigated its prophylactic potential against ethanol-induced chronic gastric injury in mice. HPLC-DAD-Q-TOF-MS analysis showed that 21 phenolic compounds were tentatively and qualitatively identified in EAH, as well as 14 phenolic compounds. Moreover, gastric ulcer indices, histopathological morphology, oxidative stress markers (MDA, GSH, SOD), inflammatory mediators (NO, PGE2), and cytokine gene expression (TNF-α, IL-6, IL-1β, iNOS) were evaluated via enzyme-linked immunosorbent assay (ELISA) and quantitative real-time PCR. Western blot was employed to assess COX-2 protein expression, while 16^S^ rRNA sequencing analyzed gut microbiota composition. The results demonstrated that EAH could play a role in gastric injury caused by long-term alcoholism by protecting gastric tissue structure, interfering with oxidative stress and inflammatory response, and remodeling the intestinal microbial community.

## 1. Introduction

Honey, a complex biological substance formed from both plant and animal origins, has been favored by consumers in recent years [1]. Although the majority of the components of honey are carbohydrates (mainly fructose and glucose), the minor components, such as proteins (e.g., Z. *jujuba*-derived protein,), enzymes (e.g., glucose oxidase, diastase), polyphenols (e.g., flavonoids, phenolic acids), organic acid (e.g., citric acid, malic acid), free amino acids (e.g., theanine), minerals (e.g., potassium, magnesium, calcium), and vitamins (e.g., vitamin C, vitamin B), also play important roles in its distinct colors, flavors, and therapeutic effects [2]. Honey is valuable because of its medical application to against various diseases, such as gastrointestinal disorders, diabetes, ulcers, and cardiovascular diseases, by improving the immune system and antibacterial, anti-inflammatory, and antioxidation properties [3,4,5].

Phytochemicals, transferred from floral nectar and pollen by bees, are regarded as the main composition enabling the medical properties of honey. These phytochemicals, including volatile compounds, phenolic compounds, carbohydrates, amino acids, and other minor components, vary among nectar-producing plant species. In particular, phenolic compounds, including phenolic acids and flavonoids, such as protocatechuic acid, 4-hydroxybenzoic acid, and rutin, have been proven to be key functional compounds that act as antioxidants and anti-inflammatories. Therefore, botanical origins influence the level and diversity of bioactive compounds and then affect the bioactive and medical properties of honey. For example, Manuka is well known for its anti-ulcer property, and Gelam honey was able to prevent airway inflammation in the ovalbumin-induced allergic asthma rat model [6,7]. Recently, research into the role of medicinal plant-origin honeys in the prevention and treatment of human diseases has intensified. Wang et al. [8] proved that the monofloral honey from *Prunella vulgaris* (PVH) showed the significant down-regulation in the disease activity index and colonic histopathological changes in dextran sulfate sodium (DSS)-induced acute colitis rats, while rosmarinic acid was regarded as the potential marker for PVH. The monofloral honey collected from the nectar of *Carthamus tinctorius* L., a traditional medicinal plant in China, exhibited significant anti-inflammatory and antioxidant effects in lipopolysaccharides-stimulated cells by inhibiting NF-κB signal transduction and activating Nrf-2/HO-1 signaling [9].

*Elaeagnus angustifolia* L., a large shrub belonging to the genus Elaeagnus of the *Elaeagnacea* (Araliaceae) family, is widely distributed in the northern regions of Asia and Europe [10]. In China, it is mainly planted in the northwest, including Ningxia, Gansu, and Xinjiang provinces, for wind break and sand fixation [11]. However, *Elaeagnus angustifolia* L. is also well known as a traditional medicinal plant, of which different parts have been used to treat a variety of illnesses and their symptoms [12]. *Elaeagnus angustifolia* L. fruit, characterized by reddish-brown and elliptic-shaped, has been applied to cure ulcers, relax muscles, kill pains, and ease fever [13]. The flowers are small, fragrant, and a yellowish-white color, and are used to treat tetanus in traditional medicine. The flowers, fruits, leaves, and bark of *Elaeagnus angustifolia* have been used in Traditional Chinese Medicine (TCM) to treat spleen–stomach asthenia, dyspepsia, enteritis, diarrhea, and cough with yellow sputum, as its pulp contains antioxidant flavonoid glycosides (e.g., quercetin and isorhamnetin derivatives). These compounds exhibit antioxidant activity and anti-inflammatory effects [14]. Phytochemical studies have revealed that the phenolic compounds, including 4-hydroxybenzoic acid, caffeic acid, kaempferol, quercetin, and luteolin, could be regarded as the major bioactive constituents responsible for these beneficial activities [15,16]. We observed that, in Xinjiang Province, China, *Elaeagnus angustifolia* L. is also a local typical nectar plant for honeybees, resulting in a favored honey with a special aroma and flavor. However, scant information is available in terms of its chemical composition and therapeutic effects. Characterizing the phenolic compounds and nutritional properties would promote the development and commercial value of EAH. Characterizing the phenolic compounds and nutritional properties is essential in understanding the mechanisms behind honey-mediated health benefits.

Taken together, in the current study, we investigated whether the consumption of EAH could protect gastric tissue, inhibit the oxidative stress and inflammatory response, and modulate intestinal microbial composition. The results of this paper would compensate for the currently scarce knowledge about the physicochemical and biochemical characterization of EAH, supporting its further utilization as a natural health-promoting food.

## 2. Materials and Methods

### 2.1. Chemicals and Materials

β-mercaptoethanol, sodium dodecyl sulfate, acrylamide, N, N′-methylenebisacrylamide, tetramethylethylenediamine, isopropanol, tris base, bromophenol blue, phenylmethylsulfonyl fluoride, and ammonium persulfate were purchased from MP Biomedicals (Santa Ana, CA, USA). COX-2 was obtained from Arigo Biolaboratories (New Taipei City, Taiwan, China). Other reagents were obtained from Tianjin Kemiou Chemical Reagent Co. (Tianjin, China).

The *Elaeagnus angustifolia* honey (EAH) samples were collected from local beekeepers in Xinjiang Province, China, specifically concentrated in the Bayingolin Mongol Autonomous Prefecture and Aksu regions. Raw EAH samples were subjected to simple filtration and jarring, then stored at 0–4 °C prior to experimental use. The collection spanned a three-year period (2018–2020) to account for potential interannual variability.

Pollen analysis was carried out according to Louveaux et al. (1978) [17] as follows: 5.0 g of honey was fully dissolved in 10 mL of distilled water and centrifuged at 2500 rpm for 10 min. The resultant sediment was evaluated under a microscope, and pollen content was expressed as the ratio of the number of pollen grains from one nectar plant to that from all nectar plants. Simultaneously, the pollen shape of the sample was identified using a scanning electron microscope (Nikon Instruments Co., Ltd., Shanghai, China).

### 2.2. Physicochemical Parameters of EAH

The physicochemical parameters of EAH, including moisture, fructose, glucose, sucrose, protein, pH, conductivity, fructose and glucose, and zinc were determined by the Association of Official Analytical Chemists (AOAC, 2005), Chinese National Standards, and Chinese entry–exit inspection and quarantine industry standards [18,19,20,21]. The quantitative determination of zinc (Zn) in EAH was performed using inductively coupled plasma mass spectrometry (ICP-MS) according to the method described by Chen et al. [22], with modifications adapted to the specific sample matrix.

### 2.3. Characterization and Quantification of Phenolic Compounds in EAH

The phenolic components in EAH samples were enriched using a solid-phase extraction (SPE) methodology [23]. Specifically, 20 g of the sample was dissolved in 100 mL of distilled water (pH adjusted to 2.0 with hydrochloric acid) and loaded onto XAD-2 (Rohm & Haas, Philadelphia, PA, USA) resin under wet conditions. Strongly polar components such as saccharides were removed by eluting with 200 mL of distilled water (pH 2.0). Subsequently, the system was rinsed with 250 mL of distilled water until neutralization, followed by elution with methanol. The methanol eluate was collected and concentrated to dryness under reduced pressure. The residue was reconstituted in 4 mL of chromatographic-grade methanol and filtered through a 0.45 µm organic-phase microporous membrane prior to chromatographic and mass spectrometric analysis.

The bioactive components detected in this research were tentatively identified using chemical standards with an in-house MS/MS database, accurate comparisons between experimental ions, calculated ions (Calc), fragment ions, and references in negative and/or positive ion modes. The collected data were analyzed for compound identification using Progenesis QI software (v3.0, Waters Corporation, Milford, MA, USA). After peak alignment, extraction, and deconvolution of the mass spectrometry data, the processed data were compared with the MetaScope database. Meanwhile, polyphenol components in EAH samples were qualitatively characterized by integrating literature with ion spectral information from the Human Metabolome Database (HMDB) and the ChemSpider and Mass Bank of North America databases. Quantitative analysis of phenolic components was performed using a standard linear regression equation derived from the extracted ion chromatogram (EIC) of HPLC-DAD-Q-TOF-MS data.

The quantitative analysis of polyphenols in EAH was performed on an Agilent 1200 Series Rapid Resolution HPLC system and the Agilent 6510 ESI-Q-TOF-MS (Agilent Technologies, Santa Clara, CA, USA). The HPLC conditions and the MS parameters refer to our previous study (Jin et al., 2017) [24]. This instrument was equipped with an Agilent Poroshell 120 EC-C18 column (2.1 mm × 100 mm, 2.7 µm). The mobile phase was A (water) and B (methanol) using the following gradient elution: 0–2 min, 85% A; 2–10 min, 85–70% A; 10–25 min, 70–10% A; 25–30 min, 10% A; 30–31 min, 10–85% A; 31–45 min, 85% A. The flow rate was set at 0.200 mL/min throughout the gradient. The injection volume was 2 µL.

### 2.4. Animal Experiments

All animal experiments were ethically approved and complied with the requirements of the Animal Ethics Committee of Northwest University. First, 18–22 g male mice provided by the Xi’an Jiaotong University School of Medicine (license: SCXK (shaan) 2017-001) were housed in a room with a controlled environment with a stable temperature (25 ± 2 °C), relative humidity (50 ± 10%), and a 10–14 h natural light cycle. During the acclimatization phase, they had free access to food and water. They were allowed to acclimatize for 1 week to the laboratory conditions before experimenting; then, the mice were separated into 4 groups (*n* = 12). All mice were allowed ad libitum access to food and water throughout the 4-week experimental period, and were randomly assigned to groups as follows:

Control group: Mice received daily oral gavage of distilled water (5 mL/kg body weight, BW).

Model group: Animals were administered distilled water (5 mL/kg BW, once daily). To establish an ethanol-induced gastric injury model, 50% (*v*/*v*) ethanol aqueous solution was additionally given at 5 mL/kg BW through oral gavage three times weekly (on Tuesday, Thursday, and Saturday), 1 h post distilled water administration.

Low-dose group: EAH solution (5 g/kg BW) was orally delivered daily, followed by ethanol challenge (50% *v*/*v*, 5 mL/kg BW) with identical frequency and temporal pattern as the model group.

High-dose group: Treatment protocol mirrored low-dose group, except using the 15 g/kg BW honey solution.

All animals were euthanized via cervical dislocation after 12 h fasting (with ad libitum water access) following the final ethanol challenge.

### 2.5. Histopathological Analysis of Gastric Tissue

For the histological assessments, sections of fresh liver and epididymal adipose were first excised and soaked overnight in 10% neutral buffered formalin, followed by embedding the tissues in paraffin and sectioning (5 µm). For general morphology, slides were stained with hematoxylin and eosin (H&E), and the prepared tissues were then observed using an optical microscope (Nikon TE2000-U). The semiquantitative scoring details were referenced to evaluate the hepatocyte.

After euthanasia by cervical dislocation, the stomachs of mice were dissected and collected. The whole stomach was washed with 4 °C normal saline solution three times to clean away the blood. After washing away, the stomach tissue was incised parallelly along the longest diameter of the lesser curvature from the gastric fundus to the pylorus. Subsequently, the gastric body was everted and thoroughly rinsed with 4 °C normal saline to remove internal residual contents. The ulcer index (UI) was scored by a person unaware of the experiment protocol in the glandular portion. The method was performed according to the GUTH method [25] with minor modifications. The parameters for the determination of UI were the color of the mucosa, hyperemia, hemorrhagic spots, and number of ulcers (0 = no lesions; 1 = small ulcers (1–2 mm), 2 = middle ulcers (3–4 mm); 4 = big ulcers (5–6 mm), 8 = many large ulcers (>6 mm). The ulcer index for each treatment group was expressed as the ratio of the total value of the ulcer score per mouse in the group to the number of mice. For routine histologic processing, the stomach tissue was dissected into two parts; one part was fixed in 10% neutral formalin solution, followed by embedding the tissues in paraffin and sectioning. For general morphology, slides were stained with hematoxylin and eosin (H&E) and periodic acid–Schiff staining (PAS), and the prepared tissues were then observed using an optical microscope (Nikon TE2000-U). Another part of the gastric tissue was soaked in 4 °C saline, ground into a homogeneous mixture, and then processed to prepare a 10% (*w*/*v*) gastric homogenate, which was then centrifuged at 2500 rpm for 10 min; the resulting supernatant was collected for subsequent assays.

### 2.6. Measurement of Inflammatory Mediators and Gene Expression Analysis

Catalase (CAT), superoxide dismutase (SOD), glutathione (GSH), malondialdehyde (MDA), nitric oxide (NO), tumor necrosis factor-alpha (TNF-α), ProstaglandinE2E2 (PGE2), and tissue protein content in gastric homogenate were analyzed using commercial kits (Nanjing Jiancheng Bioengineering Institute, Nanjing, China).

The total RNA of homogenized tissues was separated using the TaKaRa RNA separation kit (TaKaRa Biotech, Kusatsu, Japan). The cDNA templates were synthesized from equal amounts of total RNA using PrimeScipt™ RT Master Mix (TaKaRa Biotech, Japan). The genes of inducible nitric oxide synthases (iNOS), interleukin-6(IL-6), and interleukin-1beta (IL-1β) were determined using qPCR. The forward and reverse sequences of the qPCR primers were designed as follows: iNOS: 5′-CAACAGGAACCTACCAGCTCACT-3′ and 5′-AGCCTGAAGTCATGTTTGCCG-3′. IL-6: 5′-TGGAGTTCTTCTCTCTTG-3′ and 5′-TTCATATGCCAGTTCG-3′; IL-1β: 5′-GGGATGATGACGACCTGC-3′ and 5′-CCACTTGTTGGCTTATGTT-3′. The cDNA templates were amplified with SYBR Green using a quantitative real-time PCR instrument (Xi’an Tianlong Science and Technology Co., Xi’an, China). The values of gene expression were normalized with β-actin and calculated using the 2^−∆∆Ct^ formula.

### 2.7. Western Blot Analysis

Protein expression levels were quantified through Western blotting according to the protocols previously studied [26]. Stomach (50 mg) was lysed to extract proteins and determine the concentration (BCA method, Beyotime, Nanjing, China), which were separated in 10% SDS-PAGE gel according to the molecular weight of the proteins and then were transferred to polyvinylidene difluoride (PVDF) membranes (transfer conditions: current 130 mA, voltage 60 V, transfer time 90 min). After using 5% non-fat milk in TBST blocking, overnight incubation with primary antibodies against COX-2 (1:1500) (Arigo Biolaboratories Corp., Hsinchu City, Taiwan, China) and β-actin (1:15,000) (Wanleibio Technology, Shenyang, China) using secondary antibodies (HRP-conjugated Goat Anti-Rabbit IgG (H + L) 1:10,000, Proteintech, SA00001-2; HRP-labeled Goat Anti-Mouse IgG (H + L) 1:5000, Beyotime, A0216) was conducted for 1 h. The gel imaging and densitometric analyses of protein bands were subsequently performed.

### 2.8. Gut Microbiota Analysis via 16S Sequencing

The genomic DNA was extracted from rat colonic contents using the TIANamp Stool DNA Kit (DP328, TIANGEN Biotech, Beijing, China). The quality of the DNA was detected by agarose gel electrophoresis, and DNA was quantified using a Nanodrop spectrophotometer (Thermo Fisher Scientific, Waltham, MA, USA) [27]. The V3-V4 hypervariable regions of each sample 16S rRNA gene were amplified via PCR using 5′-ACTCCTACGGGAGGCAGCA-3′ and 5′-GGACTACHVGGGTWTCTAAT-3′. The PCR sequence was 98 °C for 2 min, followed by 25 cycles at 98 °C for 15 s, 55 °C for 30 s, 72 °C for 30 s, and a final extension at 72 °C for 5 min. Target fragments were cut and then recovered using a gel recovery kit (Axygen, Union City, CA, USA). Next, the amplified mixture was purified and quantified. The purified amplicons were paired-end sequenced (2 × 300) on the Illumina MiSeq platform (Illumina, San Diego, CA, USA). The sequencing data were processed through the QIIME pipeline (v1.8.0) with VSEARCH for chimera checking and removal, followed by the clustering of operational taxonomic units (OTUs) at a 97% similarity threshold. Concurrently, rarefaction curves and alpha diversity indices were calculated [28]. Linear Discriminant Analysis Effect Size (LEfSe) was employed to assess taxonomical composition across hierarchical levels and perform statistical analysis [29].

### 2.9. Statistical Analysis

All the data were determined in triplicate, and the results were shown as the mean ± standard deviation. One-way analysis of variance (ANOVA) was performed using SPSS 24.0 software at *p* < 0.05. The data were graphically presented using Origin 2025 software. Different letters indicate significant differences between any two groups.

## 3. Results

### 3.1. Characteristic Bioactive Components in EAH

**Figure 1:**
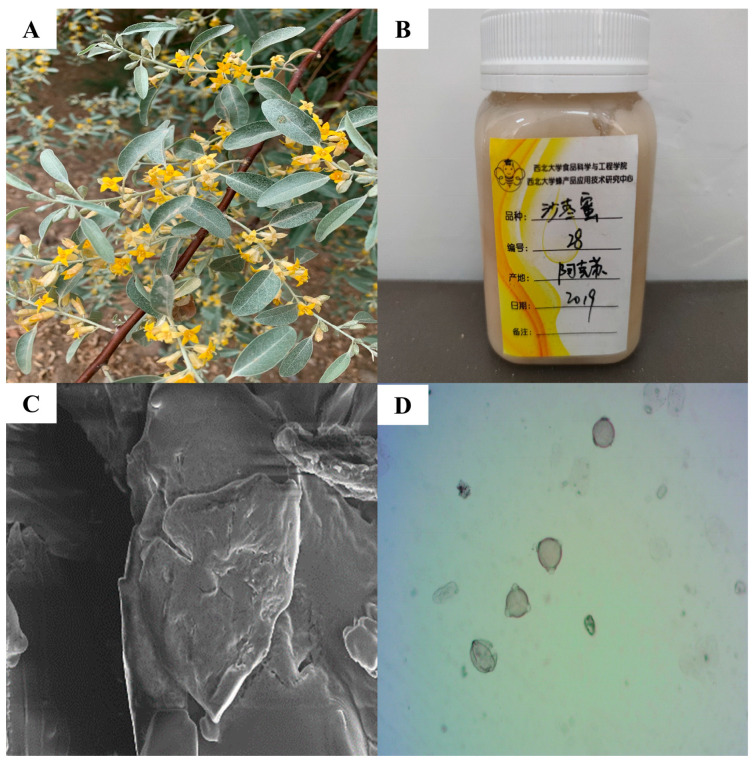
(**A**): *Elaeagnus angustifolia* flowers; (**B**): *Elaeagnus angustifolia* honey, displayed in College of Food Science and Engineering, Apicultural Product Application Technology Research Center, Northwest University.Honey variety: *Elaeagnus angustifolia* honey (originating from Aksu, Xinjiang, China); (**C**): microscope photomicrographs of pollen grains of *Elaeagnus angustifolia* honey (EHT = 5.00 kV, WD = 7.7 mm, Signal A = InLens, Mag = 3.00 KX); (**D**): scanning electron micrographs of pollen spores of *Elaeagnus angustifolia* honey.

The physicochemical parameters of honey are the basic means used to evaluate its quality. The EAH used in this study was found to contain 18.65% moisture, 34.49 g/100 g glucose, 33.53 g/100 g fructose, 68.02 g/100 g fructose and glucose, 0.90 g/100 g sucrose, 0.395 mg/kg zinc, and 1318.86 mg/kg protein; its conductivity was 0.31 mS/cm and pH was 4.16. In honey-related quality standards, there are explicit regulations on moisture content. The Codex Alimentarius Committee (2001) [30] stipulates that the moisture content in honey must not exceed 20%, and the maximum allowable sucrose content is set at 5 g/100 g. Electrical conductivity requirements are specifically defined by the FAO, stipulating a maximum of 0.8 mS/cm for nectar honeys. While there are no explicit requirements for pH ranges in honey-related quality standards, the typical pH range for common honey varieties falls between 3.00 and 5.00. Both China’s National Standard for Honey and the Codex Alimentarius mandate that the combined content of fructose and glucose in honey must be no less than 60 g/100 g, and the maximum permissible level of zinc (Zn) in honey is 25 mg/kg. All specified parameters align with international and national regulatory requirements. The morphology of the *Elaeagnus angustifolia* flower was shown in Figure 1A, exhibiting a distinct yellow coloration, while Figure 1B presents the EAH. The appearance of pollen spores is shown in Figure 1C,D, presented as a triangle with a gap at each of the three corners.

Furthermore, since LC-MS can identify the phenolic composition and qualitatively assess the relative strength of antioxidant capacity, we investigated 21 phenolic compounds in EAH by using HPLC-DAD/Q-TOF-MS along with their retention times (Tr), molecular formula, accurate comparisons between experimental ions, calculated ions (Calc), and fragment ions (Table 1). Figure S1 shows the total ion chromatogram (TIC) in the negative ion mode in EAH. All compounds identified in EAH were classified into phenolic acids and derivatives, flavonoids, and derivatives, and the process of the identification and fragmentation study was summarized as shown below.

**Figure 2:**
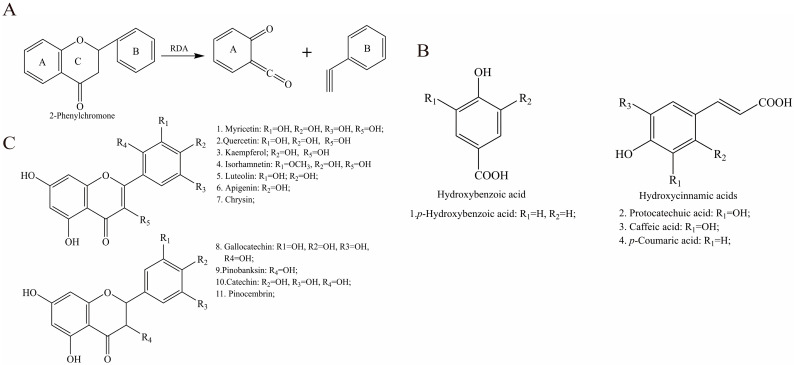
The structure of phenolic compounds: (**A**): retro Diels–Alder reaction; (**B**): flavonoids; (**C**): phenolic acid.

Phenolic acids and derivatives. A total of six phenolic acids and derivatives were characterized in EAH, through which three hydroxycinnamic acid derivatives were detected, namely, protocatechuic acid, caffeic acid, and p-coumaric acid. There is one hydroxybenzoic acid—4-hydroxybenzoic acid. The structure diagram of phenolic acids is shown in Figure 2B. Combined with diagnostic fragmentation pathway analysis and high-resolution mass spectrometry data, six phenolic acid compounds were identified in EAH, including protocatechuic acid, 4-hydroxybenzoic acid, chlorogenic acid, caffeic acid, *p*-coumaric acid, and dimethoxycinnamic acid.

Flavonoids and derivatives. Flavonoids represent a class of compounds characterized by two phenolic hydroxyl-bearing benzene rings (designated as A-ring and B-ring) interconnected through a central three-carbon chain. These compounds are structurally derived from the fundamental core of 2-phenylchromone (Figure 2A). The flavonoid aglycone core undergoes intense retro-Diels–Alder reaction (RDA) fragmentation, and thermal activation induces cleavage of the C-ring conjugated system, splitting it into A-ring-bearing dienic ions and B-ring-associated ions. This fragmentation topology is schematically reconstructed in Figure 2A, demonstrating characteristic bond rupture positions [31]. Flavonoid glycosides in -ESI mode typically generate deprotonated molecular ions [M-H]^−^ as precursor ions. These ions undergo subsequent fragmentation via the neutral loss of a glucosyl unit, yielding the dominant fragment ion [M-H-162]^−^. Further cleavage occurs through the RDA fragmentation of the C-ring, producing characteristic smaller fragment ions.

Rutin was suggested for the precursor ion at *m*/*z* 609.1456/609.1464, and produced a fragment ion [M-H]^−^ at 301 (quercetin). Gallocatechin was observed at *m*/*z* 305.0674/305.0661 and had an MS/MS fragment ion at 287, which was due to the loss of water (*m*/*z* 18.0). Gallocatechin, catechin, and isorhamnetin are also present in the fruit of the *Elaeagnus angustifolia*. Similar fragmentation ions were reported previously [32], where the major fragments were due to the neutral loss of one or more water (*m*/*z* 18.0), carbon monoxide (*m*/*z* 28.0), and carbon dioxide (*m*/*z* 44.0).

This study employed HPLC-DAD/Q-TOF-MS in ESI mode to characterize flavonoid compounds in EAH. A total of 15 flavonoid compounds were identified, primarily categorized as flavones (Figure 2A), flavanones, and flavonoid glycosides (Figure 2C). Corresponding peaks in TIC (as shown in Figure S1) were assigned as follows: Gallocatechin (No. 7), Rutin (No. 8), Myricetin (No. 9), Catechin (No. 12), Pinobanksin (No. 11), Kaempferol (No. 13), Isorhamnetin (No. 14), Luteolin (No. 15), Apigenin (No. 16), Pinocembrin (No. 17), Chrysin (No. 19), and Quercetin 3,4′-diglucoside (No. 6).

A total of 14 kinds of compounds were quantified using HPLC-DAD/Q-TOF-MS analysis. The use of EIC enabled different polyphenolic standard compounds. The information on the phenolic compound standards is shown in Table S1. The contents of the major phenolic compounds of EAH are shown in Table S2, and the identified compounds included 4 phenolic acids and 10 flavonoids. Among the phenolic acids, the two most abundant were protocatechuic acid and chlorogenic acid, with concentrations of 2.98 ± 0.87 mg/kg and 1.33 ± 0.66 mg/kg, respectively. The predominant flavonoid was rutin, reaching a content of 0.90 ± 0.06 mg/kg. In addition, rutin, pinobanksin, kaempferol, luteolin, apigenin, pinocembrin, and chrysin, identified via HPLC-DAD/Q-TOF-MS analysis, have also been confirmed as bioactive components in the fruits of *Elaeagnus angustifolia*.

### 3.2. Effect of EAH on Gastric Histological Status

#### 3.2.1. Macroscopic Evaluation of Gastric Lesions

**Figure 3:**
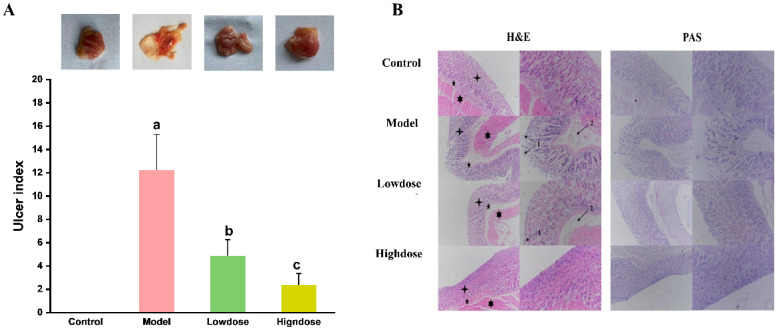
Effect of EAH on gastric histological status. The gastric lesion, gastric ulcer index (**A**), different lower case letters indicate significant differences between any two groups, and observation of gastric tissues using H & E and PAS staining (**B**). “1” indicates glands with focal loss and necrosis; “2” indicates hemorrhage; “
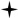
” indicates gastric mucosa; “
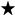
” indicates submucosa; “
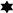
” indicates muscularis.

In this study, the ulcer index (UI) and inhibition rate of ulceration were selected as the standard to evaluate the extent of gastric lesions and the gastroprotective effect of the EAH, respectively. As shown in Figure 3, exposure to ethanol resulted in widespread gastric abrasions in the mice with the highest UI among the groups (Figure 3A). EAH effectively decreased the gastric abrasions produced by ethanol dose-dependently. The UI in the model group of mice reached a significantly elevated level of 12.25 ± 3.02, compared with the model group. The mean value of the gastric UI of the treatment group, including low-dose (60.00%) and high-dose (80.41%), was significantly decreased, while the UI inhibition rate increased significantly.

#### 3.2.2. Histological Assessment of Gastric Lesions

The effect of EAH on the gastric mucosal histopathological changes was evaluated through histologic H & E staining examinations. It can be seen from the control group (Figure 3B) that the gastric tissue glands were arranged neatly, the mucosal layer, submucosal layer, and muscle layer were complete with clear structure, and no obvious edema and inflammatory cell infiltration were observed. In the model group, it was observed that treatments of rats with ethanol caused ulcer formation with hemorrhagic spots, disrupted surface epithelium, and resulted in severe edema of the submucosal layer. In addition, the model group developed typical pathological features of chronic alcoholic gastritis, namely, the atrophied submucosal layer [33]. Compared with the model group, pretreatment of ethanol-injected mice with EAH substantially weakened the detrimental ethanol-produced gastric mucosal changes, yielding a gastroprotective effect. Among them, the high-dose group had the best outcome with a nearly typical gastric mucosal structure.

The gastric wall mucus plays an important role as a defensive barrier of the gastric epithelium against necrotizing agents such as alcohol. To investigate whether EAH protects the intact gastric wall from ethanol-induced gastric injury, PAS staining was performed on the gastric tissues to detect glycogen, mucin, and other polysaccharides [34]. The results are summarized in Figure 3B. The PAS-stained sections of the gastric tissue from a mouse in the control group showed a mucosal layer with regular glandular arrangements and dense mucin-producing cells. However, treatment of mice with ethanol caused mild or absent PAS staining, which means the depletion of gastric mucosal glycoprotein and the destruction of gastric mucosal cells. Pretreatment with EAH in ethanol-injected mice preserved gastric mucosal glycoprotein, and the tissue protection of mucosal cells from the high-dose EAH-treated groups was better.

### 3.3. Effects of EAH on Antioxidant Enzyme Activity and Oxidative Stress

**Figure 4:**
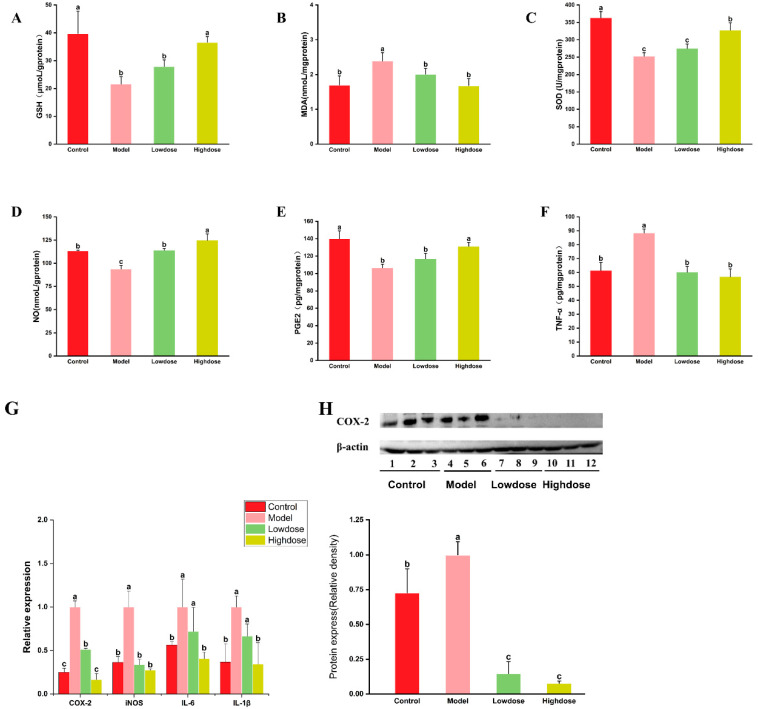
Effects of EAH on oxidative stress and levels of inflammatory markers (**A**–**C**) GSH, MDA, SOD (**D**–**F**) NO, PGE2, TNF-α (**G**,**H**). Effect of EAH on gene expression of inflammatory factors in COX-2, iNOS, IL-6, and IL-1β. Different lower case letters indicate significant differences between any two groups.

In this study, ethanol gavage resulted in a marked decrease in the level of SOD and GSH of the rats in model group, which were 252.68 U/mg protein and 21.65 μmol/g protein, respectively, whereas, compared with the control group, a significantly increase in the content of MDA was observed in this group (1.41 fold change) (Figure 4A–C). However, the ingestion of EAH effectively regulated the activity of SOD and the concentration of GSH in the gastric tissue of mice, while simultaneously suppressing the excessive accumulation of MDA. Compared to the model group, the high-dose EAH-treated group showed a significant increase in SOD activity and GSH levels by 29.59% and 69.05%, respectively, along with a notable decrease in MDA content by 29.74%.

### 3.4. Effects of EAH on Inflammatory Markers Levels and Inflammatory Cytokine Expression

Nitric oxide (NO), a free radical gas, functions as a local vasodilator in damaged gastric tissue. NO can stimulate the secretion of bicarbonate and mucus in the gastric lining and modulate the gastric acid-base environment to ameliorate gastric lesions [35]. Figure 4D shows that sustained alcohol intake resulted in a significant decrease (*p* < 0.05) in the concentration of NO within the gastric tissue of the model group, with an average concentration of 93.88 nmol/g protein, reflecting a 17.31% reduction compared to the control group. Notably, EAH supplementation can significantly increase the level of NO in the gastric tissue of the mice. Prostaglandin E2 (PGE2) plays a crucial role in mediating gastric mucus secretion. Previous studies have shown that PGE2 can maintain cellular integrity in the gastric mucosa by regulating blood flow, stimulate gastric mucus secretion, and enhance the resistance of gastric mucosal cells to ethanol-induced necrosis [36]. As shown in Figure 4E, continuous alcohol gavage for four weeks led to a significant reduction in PGE2 levels in the gastric tissue of the model group mice (*p* < 0.05), with a 23.97% decrease compared to the control group. The administration of EAH effectively mitigated the decline in PGE2 levels. Notably, the high-dose EAH treatment group exhibited a significant increase in PGE2 content in the gastric tissue, with no statistically significant difference observed compared to the control group (*p* > 0.05). Tumor necrosis factor-alpha (TNF-α) is one of the most common cytokines produced during inflammatory responses. Overexpression of TNF-α is often associated with systemic inflammatory reactions following severe conditions such as sepsis and gastritis. The concentration of TNF-α in the gastric tissues of mice from each treatment group was measured using an enzyme-linked immunosorbent assay (ELISA). As a result (Figure 4F), the TNF-α concentration in the model group was 1.44 times higher than that in the control group. The intake of EAH significantly suppressed the ethanol-induced increase in TNF-α concentration (*p* < 0.05). However, there was no significant difference between the low-dose and high-dose groups (*p* > 0.05).

The gene expression of key inflammatory factors in chronic alcoholic gastritis was quantitatively analyzed using quantitative real-time PCR (q-PCR). As shown in Figure 4G, the gene expression levels of cyclooxygenase-2 (COX-2), inducible nitric oxide synthases (iNOS), interleukin-6 (IL-6), and interleukin-1β (IL-1β) in gastric tissues of the model group were significantly higher than those in the control group, with values of 4.00-fold, 2.75-fold, 1.77-fold, and 2.72-fold, respectively. The administration of the EAH extract significantly modulated the gene expression of COX-2, iNOS, IL-6, and IL-1β (*p* < 0.05), and the regulatory effect was dose-dependent. In the high-dose group, the gene expression levels of these four inflammatory factors showed no significant difference compared to the control group (*p* > 0.05).

The expression of inflammatory factors at the protein level was evaluated using Western blotting to demonstrate the regulatory effect of EAH on the inflammatory response in ethanol-induced gastric injury. Due to experimental and sample limitations, COX-2, which showed the most active gene expression, was selected for protein-level analysis. The expression of COX-2 in gastric tissues of mice was determined after protein extraction, and the results are shown in Figure 4H, which indicate that continuous alcohol intake for 4 weeks significantly enhanced COX-2 protein expression in the gastric tissues of mice, while COX-2 was almost undetectable in the EAH-treated groups (low-dose and high-dose groups). An analysis of the column chart of relative optical density revealed that the relative expression of the COX-2 protein (using β-actin as a control) in the gastric tissues of the model group with long-term alcohol intake was 1.37 times higher than that in the control group. Low-dose and high-dose EAH intake reduced COX-2 protein expression by 85.27% and 92.25%, respectively, compared to the model group, with no significant difference between the high-dose and low-dose groups (*p* > 0.05). Although COX-2 protein was expressed to some extent in the gastric tissues of the control group, EAH intake resulted in extremely low expression, approaching zero expression. These results suggested that EAH can suppress the ethanol-induced overexpression of COX-2 in gastric tissues, thereby inhibiting ethanol-induced gastric inflammation.

### 3.5. Effects of EAH on Gut Microbiota Composition

The effects of alcohol on the gut microbiota of mice and the ameliorative effects of EAH were evaluated using 16S rRNA sequencing.

#### 3.5.1. Alpha Diversity Analysis

**Figure 5:**
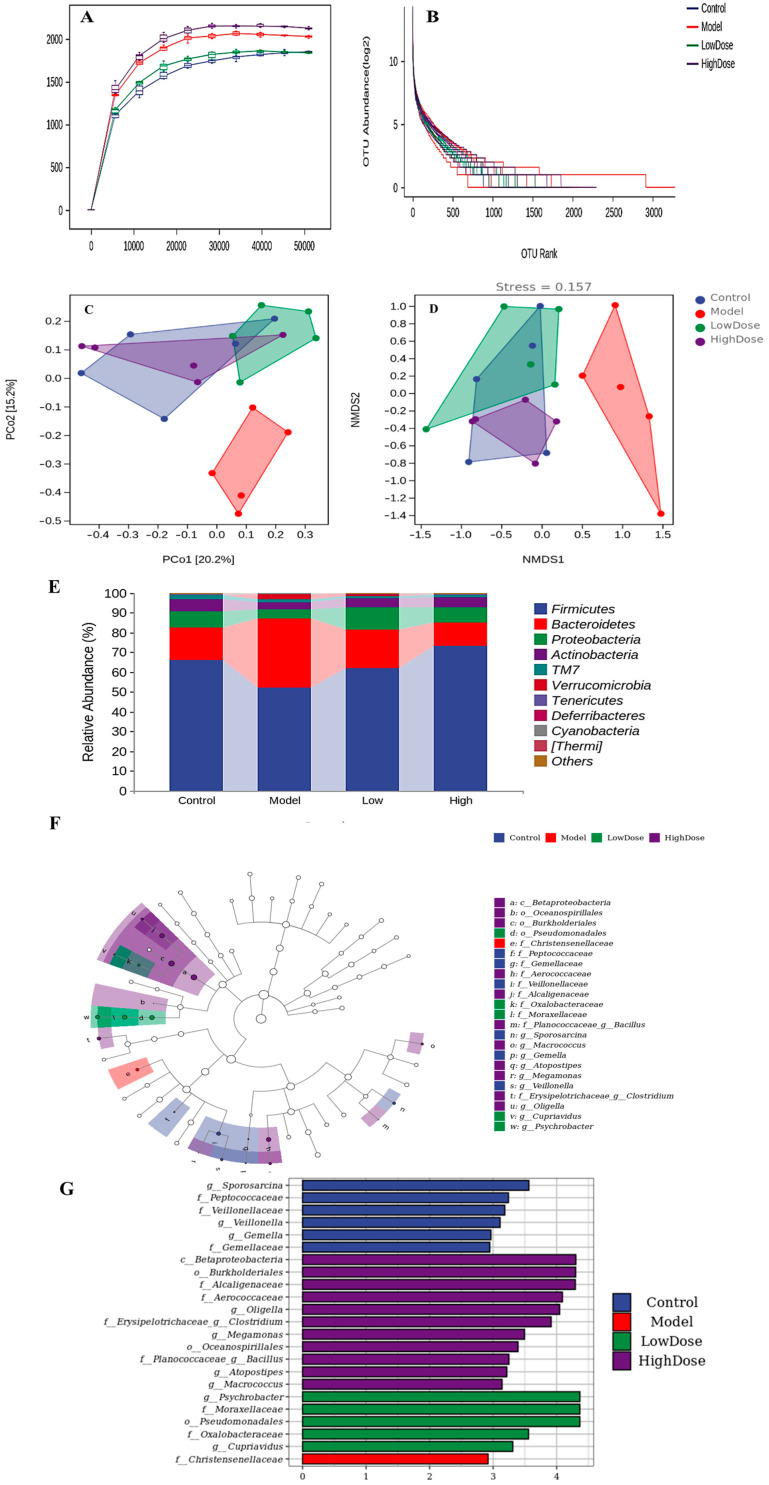
Alpha diversity analyses. (**A**): rarefaction sparse curve; (**B**): abundance grade curve; (**C**): PCoA analysis; (**D**): NMDS analysis; (**E**): composition and abundance distribution of rats in different groups at the phylum level; the LEfSe analysis of rats in different groups (**F**,**G**). (**F**): LEfSe taxonomic cladogram; (**G**): LEfSe linear discriminant analysis (LDA) score.

The Alpha diversity index primarily refers to the richness, diversity, and evenness of organisms in a local uniform habitat, and its calculation is influenced by the random sequencing depth of experimental samples [37]. In this study, rarefaction curves were used to evaluate the trends in alpha diversity across different groups as a function of sequencing depth. The results are summarized in Figure 5A. The rarefaction curves gradually plateaued with an increase in the number of randomly selected sequences, indicating that the sequencing results sufficiently captured the diversity of the gut microbial communities in each treatment group. In contrast to the rarefaction curves, rank–abundance curves were employed to visualize the distribution of both high-abundance and rare OTUs within the microbial communities. As depicted in Figure 5B, each line represented the OTU abundance distribution of a sample. The flattening of the curves with increasing OTU richness suggested a relatively even distribution of microbial community composition across the treatment groups.

The results of the gut microbial community diversity analysis in mice with chronic ethanol-induced gastric injury are shown in Table 2. Compared to the control group, long-term alcohol consumption significantly reduced the richness (Chao1 and ACE) and diversity (Shannon and Simpson) of the gut microbial communities in the model group. While the ACE index did not show a significant difference, the Chao1, Shannon, and Simpson indices were significantly lower than the control group (*p* < 0.05), which is also a typical manifestation of alcohol’s changes in gut microbiota diversity. [38]. Similarly, alcohol had a negative impact on the phylogenetic diversity and evenness (Pielou’s evenness index) of the gut microbial communities, although these differences were not significant. The intake of EAH can improve the effect of alcohol on the intestinal microbiota of mice, in the high-dose EAH treatment group, the Chao, Shannon, and Simpson indices of the gut microbial communities were significantly higher than those in the model group (*p* < 0.05) and showed no significant difference compared to the control group (*p* > 0.05). The alpha diversity results indicated that EAH effectively ameliorated the ethanol-induced reduction in gut microbial diversity.

#### 3.5.2. Beta Diversity Analysis

The multidimensional microbial data underwent dimensionality reduction using principal coordinate analysis (PCoA) and nonmetric multidimensional scaling (NMDS). Subsequent classification analysis of Beta diversity in intestinal microbiota among treatment groups was performed by examining sample distributions along ordination axes. As shown in Figure 5C (PCoA score plot), partial overlap was observed among the control, low-dose, and high-dose groups, while all three groups exhibited distinct separation from the model group. Similarly, the NMDS ordination diagram (Figure 5D) revealed closer spatial proximity of samples within the control, low-dose, and high-dose groups, indicating a high similarity coefficient of the microbiota within each group. Although the samples in the model group are dispersed, they are all distributed in the right region and are clearly distinguishable from the other three groups. The reliability of NMDS interpretation was validated by a stress value of 0.157, below the empirical threshold of 0.2 for meaningful dimensionality representation. This stress metric inversely correlates with model discrimination efficacy, where lower values denote superior ordination performance. It can be concluded that alcohol can significantly change the Beta diversity of mice intestinal microbiota, and EAH can effectively improve the effect of alcohol on the Beta diversity of mice intestinal microbiota, making it close to the normal value.

#### 3.5.3. Taxonomic Composition Analysis

The results of the taxonomic composition showed that there was an average of nine phyla with 62, 50, 56, and 63 genera per sample in the control group, the model group, the low-dose group, and the high-dose group of EAH, respectively. The composition and relative abundance of the intestinal microbial community of mice in each treatment group at the level of phylum were shown in Figure 5E, which mainly includes more than ten phyla, such as *Firmicutes*, *Bacteroidetes*, *Proteobacteria*, and *Verrucomicrobia*. Among them, *Firmicutes*, *Bacteroidetes*, and *Proteobacteria* are the major components. In comparison, the relative abundance of *Firmicutes* in the model group was only 51.68%, which was lower than that in the control group, while the relative abundance of *Bacteroidetes* and *Verrucomicrobia* increased compared with that in the control group, reaching 38.91% and 2.24%, respectively.

This suggests that the intake of EAH can ameliorate the effects of alcohol on the intestinal microorganisms of mice, and the abundance of *Firmicutes* in the high-dose group was 73.30%, which was significantly higher compared with the model group. The relative abundance of *Bacteroidetes* and *Verrucomicrobia* in the high-dose group was lower than that of the model group, with 12.07% and 0.01%, respectively.

In order to determine the differential strains of mice gut microbiota in different treatment groups, this experiment was performed using the LEfSe (LDA Effect Size) analysis of differences at all classification levels simultaneously in order to find robust differences and biomarkers between treatment groups. Figure 5F,G presents the LEfSe results of all samples. Figure 5F depicts a taxonomic cladogram, which visualizes the phylogenetic hierarchy of biomarker taxa across all samples. The control, model, low-dose, and high-dose groups are color-coded in blue, red, green, and purple, respectively. The concentric circles correspond to taxonomic ranks from phylum (innermost) to genus (outermost), with node sizes reflecting the average relative abundance at each taxonomic level. Node sizes are scaled according to the mean relative abundance of taxa at each taxonomic rank. Figure 5G illustrates the LDA score distribution histogram, highlighting significantly enriched taxa and their effect magnitudes in respective groups. Table S3 summarizes the relative abundance (only significantly upregulated entries were shown), LDA scores, and *p*-values for differential taxa. LDA score ≥ 2 indicated a taxon’s elevated relative abundance in the corresponding group, while *p* < 0.05 confirms significant differences compared to other treatments. The results indicated that there were no significant differences in the gut microbiota among the groups of mice at the phylum level. At the class level, the high-dose group exhibited a significant difference in betaproteobacteria compared to the other three groups (*p* < 0.05). At the order level, *pseudomonadales* in the low-dose group and *oceanospirillales* in the high-dose group showed significant differences from the other groups of mice (*p* < 0.05). At the family level, *Christensenellaceae* in the model group and *oxalobacteraceae* in the low-dose group were identified as differential bacteria (*p* < 0.05).

Notably, in addition to gastric tissue inflammation, this experiment found that long-term alcohol intake caused severe flatulence in the model group, while the intake of EAH effectively alleviated alcoholic flatulence in the mice (Figure S2).

## 4. Discussion

The last several years have seen a decrease in the incidence of gastric cancer worldwide; however, gastric cancer still remains the most common cancer and cause of mortality. Alcohol, which has easily passed through the cell membrane, induced gastric mucosal damage and is associated with mucosal injury, including mucosal bleeding, loss of mucosal integrity, and cell death, leading to the formation of a gastric ulcer. Gastric ulcers can contribute to gastric carcinogenesis, according to reports that several studies have observed gastric cancer in patients with gastric ulcers. Several drugs are used to prevent gastric ulcers; however, basically, a strategy to protect the gastric mucosa is necessary to prevent gastric ulcers [39]. In this study, EAH protected gastric mucosa via four key mechanisms: protecting gastric tissue structure, interfering with oxidative stress and inflammatory response, and remodeling the intestinal microbial community.

### 4.1. Characterization and Quantification of Composition in EAH

Precursors subjected to the MS/MS analysis produced fragment ions due to losing carbon dioxide (CO_2_) (*m*/*z* 44.0) or water molecules (*m*/*z* 18.0) in negative ion mode because polyphenols contain one or more carboxyl or hydroxyl groups. 4-hydroxybenzoic acid was suggested for the precursor ion at *m*/*z* 137.0245/137.0244, and produced a fragment ion [M-H]^−^ at 93, where the major fragments were due to the neutral loss of one CO_2_. Alongside the molecular ions, the MS/MS analysis also detected fragment ions at *m*/*z* 135 for protocatechuic acid and *m*/*z* 161 for caffeic acid. These fragment ions correspond to the [M-H-H_2_O]^−^ species formed by the loss of a water molecule (H_2_O) from their corresponding molecular ions through dehydration. In the mass spectrum of chlorogenic acid ([M-H]^−^ at *m*/*z* 353.0894), two dominant fragment ions were observed at *m*/*z* 191 and *m*/*z* 179. These fragments are caused by the neutral loss of caffeic acid and quinic acid by the loss of molecular ions, respectively. In accordance with the reports, based on structural variations, flavonoids in honey are primarily categorized into multiple classes, including flavones, flavanones, flavonols, isoflavones, and anthocyanins. Additionally, under the high-polysaccharide conditions of honey, these compounds frequently conjugate with sugars to form glycosides. Generally, flavonoids in negative electrospray ionization (-ESI) mode are predominantly characterized by molecular ion peaks [M-H]^−^ and fragment ions such as [M-H-CO]^−^ and [M-H-H_2_O]^−^. Notably, when methyl or methoxy substituents are present, additional fragment ions including [M-CH_3_]^−^ and [M-CH_3_-CO]^−^ are observed in the mass spectra.

### 4.2. EAH Enhances Gastric Mucosal Defense for Ethanol Damage

Chronic alcohol consumption has been demonstrated to induce severe gastric damage. In the present study, four-week continuous ethanol administration resulted in significant behavioral manifestations in the mice in the model group, including reduced food intake and behavioral depression, accompanied by histopathological evidence of ulcerative damage in gastric tissues. In the 1960s, the concept of gastric mucosal barriers was introduced, which indicated that the mucus secreted by the upper mucous layer of normal gastric mucosa connects with cells to form a gastric mucosal protective barrier that protects gastric tissue from the attack of aggressive factors [33]. In the astringent action hypothesis of the etiology of gastric ulcer, the primary step to prevent gastric ulcer is forming a resistant layer over the lining that preserves the intact gastric mucosa from the destruction of toxins or irritants, such as alcohol [40]. It was not difficult to see in this experiment that EAH could prevent the gastric mucosal structure of mice from ethanol injury, compared with the mice in the model groups. EAH can effectively prevent chronic ethanol-induced hemorrhagic ulcers and preserve gastric mucosal integrity in mice. The possible action mechanisms could involve the gastrointestinal protection of polyphenolic compounds, such as catechin, chlorogenic acid, protocatechuic acid, chlorogenic acid, and caffeic acid, gallic acid, and tannins exhibit cytoprotective properties and are associated with antiulcerogenic activity. Their astringent action induces the precipitation of microproteins at the ulcer site, thereby forming an impervious protective layer covering the mucosal surface. Additionally, these compounds demonstrate vasoconstricting effects, which further safeguard the underlying mucosa from damage caused by external irritants [41].

### 4.3. EAH Attenuates Oxidative Stress in Chronic Ethanol-Induced Gastric Damage

In addition to the defense barrier, the antioxidant system is also an important factor protecting the structure and function of the gastric mucosa. Previous studies reported that oxidative stress and inflammation were greatly responsible for gastric injury. Oxidative stress, which is a state of unbalanced levels of reactive oxygen species (ROS), can cause gastric mucosal injuries, including ulceration, erosion, and hemorrhage. Oxygen-derived free radicals, primarily hydroxyl radicals, superoxide anions, and lipid peroxides, are the harmful species known to cause the gastric ulcer. A major source of ROS in ethanol-injured gastric tissue is the infiltration of activated neutrophils, which will stimulate the release of several pro-oxidative enzymes and free radicals, leading to an oxidative burst.

Previous studies have shown that chronic ethanol-induced gastritis is closely related to oxidative stress dysregulation; this pathological state is attributed to chronic alcohol intake-induced infiltration of activated neutrophils into the gastric mucosa. These cells stimulate the release of multiple pro-oxidant enzymes and free radicals in gastric tissues, thereby disrupting ROS homeostasis, the derived free radicals, predominantly hydroxyl radicals and superoxide anions, and, therefore, could induce gastric mucosa injury. This gastric mucosa injury is characterized by the ulceration, erosion, and hemorrhage of the gastric mucosa, which collectively drive the pathogenesis of chronic gastritis [42,43]. At the same time, free radical-induced lipid peroxidation generates excessive malondialdehyde (MDA), which serves as a key biomarker for assessing oxidative stress in gastric tissues of alcohol-related injury models [44]. In this study, the MDA of gastric tissue in the alcohol-related gastric injury model group was approximately 1.5 times higher than that in the control group, indicating that chronic alcohol intake induces excessive production of lipid peroxidation products and consequent oxidative stress in the gastric mucosa of mice. The intake of EAH significantly suppressed lipid peroxidation in the mice’s gastric tissues, effectively reducing the excessive accumulation of MDA and maintaining lipid peroxidation at normal levels.

Under normal physiological conditions, gastric tissue cells contain a variety of antioxidants that neutralize ROS, forming the primary line of defense for gastric tissue. These antioxidants primarily include glutathione (GSH) and superoxide dismutase (SOD). GSH acts as an intracellular reducing agent and a cofactor for antioxidant enzymes; SOD exerts its antioxidant effects by converting the harmful superoxide anions in the body into less dangerous hydrogen peroxide [45]. However, when the levels of ROS exceed the normal range, persistent ROS production depletes antioxidants in gastric cells and inhibits the activity of antioxidant enzymes [42]. Four-week continuous ethanol administration resulted in model group mice exhibiting GSH levels that were only half of the control group, the activity of SOD decreased by 30.41%, indicating that the ethanol-induced overexpression of reactive oxygen species consumed GSH in the gastric tissue of the mice and inhibited the activity of SOD, ultimately compromising their antioxidant capacity. The results of the oxidative stress-related indicators in this study demonstrated that the intake of EAH significantly increased the content of GSH and the activity of SOD enzyme, demonstrating its potent antioxidant activity. These findings suggest that EAH effectively alleviates ethanol-induced oxidative stress lesions of gastric tissue caused by long-term alcohol consumption. Recent studies have demonstrated a significant correlation between the antioxidant activity of foods and their phenolic compositions. Phytochemical analysis has identified gallocatechin, catechin, and quercetin-3,4′-diglucoside as predominant phenolic constituents in EAH. These compounds, which are also recognized as the primary bioactive constituents contributing to the antioxidant capacity of *Elaeagnus angustifolia* fruits, mediate their antioxidant effects through dual mechanisms: free radical scavenging capacity and superoxide anion inhibition [13,14,46]. Therefore, phenolic compounds constitute the fundamental material basis through which EAH modulates oxidative stress damage in organisms with chronic ethanol-induced gastric injury.

### 4.4. EAH Regulates Inflammatory Factor Levels and Inflammatory Cytokine Expression in Chronic Ethanol-Induced Gastric Damage

Under physiological conditions, the levels of NO and PGE2 in gastric tissue are dependent on the extent of gastric mucosal injury. Excessive intake of high-concentration alcohol triggers an inflammatory response, which often leads to severe gastric mucosal damage, subsequently suppressing the synthesis of NO and secretion of PEG2 [47,48,49]. Therefore, PGE2 and NO are frequently regarded as biomarkers of gastric tissue anti-inflammatory response in recent studies. The results of this study demonstrated that EAH can enhance the levels of NO and PEG2 in gastric tissue, enabling the modulation of gastric mucus secretion, preserving mucosal integrity, and maintaining physiological acid-base homeostasis, which counteract ethanol-induced inflammatory injury. Previous studies have shown that high levels of TNF-α promote leukocyte adhesion to gastric mucosa, exacerbating inflammatory responses. Consequently, TNF-α has been identified as a principal mediator in ethanol-induced gastritis pathogenesis, with TNF-α’s expression levels serving as a reliable indicator for quantifying inflammatory progression in gastric mucosa [50].

Elevated levels of pro-inflammatory cytokines trigger apoptosis of gastric mucosal vascular endothelial cells, thereby inducing mucosal injury, which constitutes another primary reason for ethanol-induced gastric damage [45]. Consequently, the tissue-specific expression profiles of these cytokines serve as reliable biomarkers for assessing inflammatory severity. It has been confirmed that inflammatory cytokines associated with alcoholic gastric damage, including COX-2, iNOS, IL-6, and IL-1β, among others.

COX-2 is the most actively expressed inflammatory factor in the ethanol-intragastric administration model. COX-2 is an inducible isoform of cyclooxygenase (COX), while the other isoform of COX is cyclooxygenas-1(COX-1); both isoforms can catalyze the synthesis of Prostaglandin (PG) from arachidonic acid [51]. Under physiological conditions, COX-1 exerts constitutive functional roles in the gastrointestinal tract, synthesizing PG to maintain the integrity, and it can counteract inflammatory injury, while COX-2 exhibits negligible expression levels and minimal contribution to gastrointestinal homeostasis at low levels. However, when the gastrointestinal mucosa is damaged by pro-inflammatory factors, including TNF-α and IL-1β, or by mitogenic agents, COX-2 expression is induced. Consequently, the overexpression of COX-2 is closely associated with inflammatory responses in the gastrointestinal tract and the occurrence of cancer [52,53,54]. Under oxidative stress, overexpressed COX-2 catalyzes the conversion of PG into oxidized metabolites, notably 8-isoprostaglandin F_2alpha_(8-*iso*-PGF_2alpha_); these metabolites disrupt gastric mucosal integrity by promoting vasoconstriction and enhancing platelet aggregation, thereby exacerbating the progression of gastric injury [55]. This observation may explain why chronic alcohol intake in the model group led to a significant reduction in the content of PGE2 in gastric tissues (*p* < 0.05). The administration of EAH effectively suppressed COX-2 overexpression in gastric tissues. Notably, the levels of COX-2 in the high-dose EAH treatment group showed no significant difference compared to the control group (*p* > 0.05).

The upregulation of INOS represents a hallmark inflammatory response in ethanol-induced gastric injury models. This enzyme exists alongside two constitutive isoforms: neuronal nitric oxide synthase (nNOS) and endothelial nitric oxide synthase (eNOS), which are primarily involved in physiological regulation. All three isoforms catalyze the L-arginine conversion to NO, a key biosignaling molecule mediating both homeostatic processes and pathological cascades [56]. Notably, NO, derived from eNOS and nNOS, primarily functions as a cell signaling molecule that mediates physiological protection, playing a crucial role in the healing of gastric injury by maintaining the integrity of gastric epithelial cells, regulating mucosal blood flow, and modulating mucus secretion and synthesis. In contrast, when iNOS in macrophages is exposed to intracellular pathogens, tumor cells, or microbial products, it produces harmful NO, which exacerbates the inflammatory response at the site of gastrointestinal injury, thereby accelerating the progression of injury [57,58]. Our experimental results demonstrated that the gene expression of iNOS was significantly upregulated in the gastric tissues of the ethanol-induced mice group, showing a consistent regulatory pattern with both the acute ethanol-induced gastric injury model, consistent with previous studies. This suggests that the excessive positive expression of iNOS in the gastric tissue induced by alcohol is a typical manifestation of both acute and chronic alcoholic gastric injury. The intake of EAH significantly suppressed the overexpression of iNOS while restoring physiological NO production (*p* < 0.05), which aligns with the findings of Yang et al. [35]. on the gastroprotective role of Taraxacum extract in chronic ethanol-induced injury [35]. The results of inflammatory factor gene expression in this study indicated that EAH can control the production of harmful NO derived from excessive iNOS expression while regulating the NO content in the gastric mucosa, maintaining the content of NO at normal levels, and protecting the integrity of the gastric mucosal microcirculation from ethanol-induced damage.

IL-6 and IL-1β are among the most common inflammatory factors involved in the body’s inflammatory response [59]. IL-6 is a pro-inflammatory factor that participates in the inflammatory response by regulating the expression of genes involved in the cell cycle and inhibiting apoptotic gene expression. IL-1β is a pro-inflammatory factor regulated by the Toll-like receptor (TLR) pathway, and the activation of TLR leads to its overexpression [50,54,60]. The abuse of alcohol excessively stimulates the expression of the innate immune system, thereby hyperactivating the levels of pro-inflammatory cytokines. The activation of inflammatory mediators frequently stimulates neutrophils and causes gastric damage [61]. Therefore, IL-6 and IL-1β show significantly elevated expression in patients with ethanol-induced gastric damage, which includes ulcerative gastritis and inflammation-induced gastric carcinogenesis [50]. The results of inflammatory factor gene expression in this study indicated that the intake of EAH can significantly inhibit the excessive positive expression of IL-6 and IL-1β induced by alcohol in a dose-dependent manner. In the high-dose EAH treatment group, the positive expression of IL-6 and IL-1β genes in the gastric tissue of mice showed no significant difference compared to the normal group (*p* > 0.05). This suggested that EAH-mediated inhibition of TNF-α, IL-6, and IL-1β represents a critical preventive mechanism against chronic ethanol-induced gastric damage.

Elaeagnus angustifolia (EA) is a natural plant with anti-inflammatory phytomedicinal properties in ethnomedical practice, demonstrating efficacy in alleviating inflammatory pain, vomiting, and diarrhea-related gastrointestinal distress [62]. Under physiological conditions, COX-2 protein remains undetectable or minimally expressed in the gastric mucosa. However, chronic gastritis pathogenesis involves multifactorial interactions, including not only exogenous irritants such as ethanol and dietary carcinogens but also endogenous stressors such as psychological stress, circadian disruption, and autoimmune dysregulation, all contributing to a subclinical pro-inflammatory milieu [35]. Phytochemical analyses of EA extracts identified flavonoids as the primary bioactive constituents, which exhibit significant COX-1/COX-2 inhibitory effects comparable to indomethacin in mitigating inflammatory responses without adverse effects. This phytotherapeutic advantage highlights its potential as a natural anti-inflammatory alternative [63].

The phenolic composition analysis revealed that EAH contains many kinds of flavonoids, including isorhamnetin, catechin, apigenin, luteolin, and chrysin. These flavonoids exhibit significant anti-inflammatory activity, albeit through distinct mechanisms of action governed by their structural variations. Isorhamnetin primarily exerts antioxidant effects by modulating inflammatory mediators and cytokines [64]. Catechin, characterized by a hydroxyl group at the C3 position, regulates inflammatory processes through inhibition of enzyme activity associated with inflammatory responses [65]. Apigenin acts as a classical COX-2 inhibitor [66]. The presence of a C2-C3 double bond and hydrogen bonding at the C3 position enables luteolin and chrysin to suppress pro-inflammatory factors, thereby modulating inflammatory cascades [67]. These phenolic constituents provide a phytochemical basis for the anti-ethanol-induced gastric tissue inflammatory responses.

### 4.5. EAH Alters the Gut Microbial Structure in Chronic Ethanol-Induced Gastric Damage

The gut microbiota refers to the collective microorganisms in the gastrointestinal tract, including bacteria and other microbes, with an enormous quantity and diversity, which are numerically vast (10^13^ to 10^14^ cells) and taxonomically diverse, with over 1000 bacterial species identified to date. The majority of these species belong to the phyla *Firmicutes* and *Bacteroidetes*. These microorganisms facilitate energy harvest from dietary substrates and contribute to forming a barrier against pathogens [68]. However, the homeostasis of gut microbiota is highly susceptible to disruption by external factors such as diet, circadian rhythm, and alcohol consumption. Alcohol is one of the factors that can disrupt the gut microbiota. Previous studies have shown that chronic alcohol intake alters the abundance of gut microbiota and disturbs microbial metabolic pathways, thereby inducing alcohol-associated diseases, including alcoholic liver injury, gastric injury, and colonic [69].

The gut permanently colonized microbiota is predominantly composed of *Firmicutes* and *Bacteroidetes*, followed by *Actinobacteria*, *Proteobacteria*, *Fusobacteria*, and *Verrucomicrobia*, with methanogenic archaea and eukaryotic microorganisms also constituting minor components [68]. Relevant studies have shown that patients with ulcerative colitis exhibit a significant increase in the relative abundance of *Bacteroidetes* within the gut microbiota, suggesting a correlation between the colonization of *Bacteroidetes* in the gut and the occurrence and exacerbation of ulcerative colitis, which may serve as a potential driver in the pathogenesis of ulcerative colitis [70]. Under normal physiological conditions, the gut microbial ecosystem exists in a relatively stable state within the intestinal environment. The colonization of microbes in the gut depends critically on their adhesion ability, while surface hydrophobicity and auto-aggregation are key factors influencing this adhesion ability [71,72]. Probiotics in the gut exhibit robust adhesion ability to the intestinal mucus layer, enabling them to reside and colonize stably, thereby maintaining a balanced and healthy microbiota. However, abrupt external stimuli (such as alcohol, stress, temperature fluctuations) can alter microbial auto-aggregation capacity, leading to changes in the relative abundance of gut microbial communities and disruption of their homeostatic state [73,74,75]. Phenolic-enriched honey significantly enhances microbial surface hydrophobicity, thereby amplifying probiotic adhesion capacity and restoring microbial equilibrium in suboptimal gut conditions [76]. This mechanistically explains our experimental observation that EAH effectively counteracted ethanol-induced changes in the relative abundance of *Firmicutes*, *Bacteroidetes*, and *Verrucomicrobia* in the intestines of mice.

The *Christensenellaceae* family, belonging to the *Bacteria* domain, phylum *Firmicutes*, class *Clostridia*, and order *Christensenellaceae*, has been identified as a health-associated microorganism. Current evidence demonstrates its potential correlations with the pathogenesis of asthma, diabetes mellitus, and colitis [77,78]. The *Christensenellaceae* can ferment monosaccharides to produce hydrogen (H_2_), with a hydrogen production capacity approximately seven-fold higher than that of *B. thetaiotaomicron* under identical conditions. Furthermore, *Christensenellaceae* can establish a symbiotic relationship with *methanobrevibacter* smithii, the most abundant methanogen in the human gut, by providing hydrogen as a substrate, thereby enhancing methane (CH_4_) biosynthesis [79].

Flatulence, a common gastrointestinal symptom, is often associated with dietary indiscretions such as excessive alcohol intake or consumption of contaminated foods, though its precise etiology remains incompletely understood. Previous studies have shown gut dysmotility and the disruption of intestinal microbial homeostasis to be key etiological factors of flatulence. Following the destabilization of microbial community homeostasis, bacterial colonization drives the excessive production of H_2_ and CH_4_, while impaired intestinal peristalsis impedes timely gas evacuation, ultimately leading to pathological gas accumulation [80,81]. The above analysis results of the gut microbiota indicated that long-term alcohol intake can alter the homeostasis of the gut microbiota in mice, promoting the colonization of gas-producing bacteria, including *Christensenellaceae*, which subsequently induces excessive H_2_ and CH_4_ production and leads to severe flatulence. However, the intake of EAH effectively mitigates ethanol-induced dysbiosis, maintains physiological microbial community structure, and prevents the occurrence of gastrointestinal bloating. Studies have demonstrated that phenolic compounds in flowers of elaeagnus angustifolia, such as isorhamnetin, catechin, and gallocatechin, exhibit potent flatulence-alleviating effects by modulating microbial metabolism [62]. Meanwhile, clinical trial data indicate that the long-term intake of polyphenol-rich foods can significantly improve the state and composition of the gut microbiota and promote health-associated microbial homeostasis [82]. Therefore, the phenolic components in EAH are an important material basis for reshaping the gut microbiota in mice with chronic alcoholic gastric damage.

## 5. Conclusions

This study investigated the protective effects of EAH on chronic ethanol-induced gastric injury in mice. The gastric histopathology, oxidative stress parameters, gene expression of inflammatory cytokines, protein expression detected using Western blotting, and gut microbiota composition were systematically analyzed. The results demonstrated that EAH administration significantly reduced gastric ulcer indices and alleviated ethanol-induced mucosal damage. In terms of oxidative stress, EAH suppressed MDA accumulation while enhancing SOD, GSH, NO, and PGE2 levels. Furthermore, EAH modulated the gene expression of inflammatory factors and the synthesis of anti-inflammatory markers, while also inhibiting the overexpression of COX-2 protein. Additionally, EAH administration modulated intestinal microbial composition at the phylum level, which can increase the abundance of *Firmicutes* while reducing *Bacteroidetes* and *Verrucomicrobia* and suppressing the overcolonization of gas-producing *Christensenellaceae* at the family level. Moreover, the preventive effects of EAH against chronic ethanol-induced gastric injury could be closely related to the phenolic compounds, mainly isorhamnetin, catechin, apigenin, luteolin, and chrysin, which present a phytochemical basis for protecting and preserving gastric tissue integrity, mitigating oxidative stress, modulating inflammation, and remodeling gut microbial homeostasis. Taken together, this work proves the prophylactic protection of EAH against chronic ethanol-induced gastric injury through multi-pathway mechanisms, highlighting its potential as a therapeutic value for chronic alcoholic gastric injury.

## Figures and Tables

**Table 1:** Characterization of the phenolic compounds in EAH using HPLC-DAD/Q-TOF-MS.

NO.	Tentative Assignment	Tr ^a^ (min)	[M-H]^− b^ (*m*/*z*)	[M-H]^− c^ (*m*/*z*)	Molecular Formula	Error (ppm)	Fragment Ions (*m*/*z*)
1	Protocatechuic acid	3.86	153.0194	153.0194	C_7_H_6_O_4_	0.65	153; 135; 109
2	4-Hydroxybenzoic acid	5.48	137.0245	137.0244	C_7_H_6_O_3_	0.45	119; 93
3	Chlorogenic acid	7.45	353.0894	353.0878	C_16_H_18_O_9_	4.53	191; 179
4	Caffeic acid	8.76	179.0355	179.0350	C_9_H_8_O_4_	2.70	161; 135
5	*p*-Coumaric acid	12.16	163.0404	163.0401	C_9_H_8_O_3_	2.26	119
6	Quercetin 3,4′-diglucoside	13.43	625.1434	625.1405	C_27_H_30_O_17_	4.64	463; 301
7	Gallocatechin	15.17	305.0674	305.0661	C_15_H_14_O_7_	4.26	287; 179; 161; 137
8	Rutin	15.74	609.1456	609.1464	C_27_H_30_O_16_	−0.82	301
9	Myricetin	16.61	317.0321	317.0303	C_15_H_10_O_8_	5.68	178; 151; 137
10	Dimethoxycinnamic acid	17.80	207.0688	207.0668	C_11_H_12_O_4_	9.66	163
11	Pinobanksin	18.49	271.0629	271.0612	C_15_H_12_O_5_	6.27	243; 227; 151;
12	Catechin	18.51	289.0710	289.0719	C_15_H_14_O_6_	−3.11	271; 257; 161; 151;
13	Kaempferol	18.89	285.0418	285.0405	C_15_H_10_O_6_	4.56	267; 257; 241; 151
14	Isorhamnetin	19.65	315.0519	315.0510	C_16_H_12_O_7_	2.70	315; 300; 151;
15	Luteolin	19.79	285.0409	285.0405	C_15_H_10_O_6_	1.71	257; 151;
16	Apigenin	20.03	269.0462	269.0455	C_15_H_10_O_5_	2.60	269; 251; 225; 151
17	Pinocembrin	21.47	255.0665	255.0663	C_15_H_12_O_4_	0.73	237; 227; 221; 151;
18	3-o-acetylpinobanksin	21.65	313.0719	313.0718	C_17_H_14_O_6_	0.32	313; 269
19	Chrysin	22.12	253.0518	253.0506	C_15_H_10_O_4_	4.74	253; 235; 151;
20	Caffeic acid phenylethyl ester	22.19	283.0982	283.0976	C_17_H_16_O_4_	2.12	283; 253; 151;
21	Galangin	22.61	269.0463	269.0455	C_15_H_10_O_5_	2.97	269; 251; 225;

^a^ Retention times detected using HPLC-DAD/Q-TOF-MS (min); All compounds were tentatively identified; ^b^ Experimentally observed molecular ions of compounds under HPLC-DAD/Q-TOF-MS detection in negative ESI mode; ^c^ Theoretical molecular ions of compounds under HPLC-DAD/Q-TOF-MS detection in negative ESI mode.

**Table 2:** Microbial diversity index of the rats in different groups.

	Chao1	ACE	Shannon	Simpson	Faith	Pielou
Control	1927 ± 287 ^a^	1602 ± 273 ^a^	7.44 ± 0.92 ^a^	0.97 ± 0.02 ^a^	104 ± 11 ^a^	0.66 ± 0.07 ^a^
Model	1326 ± 180 ^b^	1529 ± 539 ^a^	6.18 ± 0.41 ^b^	0.92 ± 0.02 ^b^	100 ± 17 ^a^	0.62 ± 0.06 ^a^
Lowdose	1594 ± 246 ^b^	1585 ± 248 ^a^	6.61 ± 0.48 ^b^	0.94 ± 0.04 ^b^	105 ± 13 ^a^	0.64 ± 0.05 ^a^
Highdose	2249 ± 301 ^a^	1650 ± 304 ^a^	7.80 ± 0.31 ^a^	0.97 ± 0.01 ^a^	107 ± 6 ^a^	0.69 ± 0.04 ^a^

The data in the table were shown as the mean *±* standard deviation. Different lowercase letters indicated significant differences between any two groups.

## Data Availability

The original contributions presented in this study are included in the article/Supplementary Materials. Further inquiries can be directed to the corresponding authors.

## Supplementary Materials

The following supporting information can be downloaded at: https://www.mdpi.com/article/10.3390/foods14091600/s1, Figure S1: Total ion chromatogram in the negative ion mode of EAH; Figure S2: The flatulence of rats’ gastrointestinal systems in different group; Table S1: Table information of the phenolic compound standard; Table S2: Contents of the 14 polyphenols in EAH; Table S3: Different species of gut microbiota of rats in different groups. (Relative abundance > 2, *p* < 0.05)
