# Supplementary material for: Effect of Elaeagnus angustifolia Honey in the Protection Against Ethanol-Induced Chronic Gastric Injury via Counteracting Oxidative Stress, Interfering with Inflammation and Regulating Gut Microbiota in Mice
Source: Foods. 2025 May 1;14(9):1600. doi: 10.3390/foods14091600 (PMC12072024; doi:10.3390/foods14091600)
Supplement: Supplementary file 1 [file foods-14-01600-s001.zip › foods-3585536-supplementary.pdf]

Supplementary material

Table S1. Table information of the phenolic compound standard

NO.	Compounds	Tr (min)	[M-H]-	Standard curve	R^2	Linear range (μg/mL)
1	Protocatechuic acid	4.12	153.0193	y=4E-07x-0.2973	0.9991	1.0-50.0
2	Chlorogenic acid	7.50	353.0878	y=5E-07x-0.0695	0.9994	1.0-10.0
3	Caffeic acid	8.75	179.0350	y=1E-07x-0.1610	0.9998	0.1-10.0
4	*p*-Coumaric acid	12.09	163.0401	y=4E-07x-0.1453	0.9993	0.1-10.0
5	Rutin	15.70	609.1461	y=6E-07x-0.2345	0.9990	1.0-10.0
6	Myricetin	16.61	317.0303	y=8E-08x+0.0854	0.9996	1.0-10.0
7	Kaempferol	18.91	285.0405	y=4E-08x-0.0482	0.9997	1.0-10.0
8	Luteolin	19.78	285.0405	y=2E-08x+0.0524	0.9999	0.1-10.0
9	Apigenin	20.02	269.0455	y=4E-08x-0.1111	0.9998	0.1-10.0
10	Pinocembrin	21.49	255.0663	y=3E-08x-0.1022	0.9998	0.1-10.0
11	3-o-acetylpinobanksin	21.62	313.0718	y=4E-08x-0.109	0.9998	0.1-10.0
12	Chrysin	22.04	253.0506	y=3E-08x-0.0584	0.9999	0.1-10.0
13	Caffeic acid phenylethyl ester	22.17	283.0976	y=2E-08x-0.0834	0.9998	0.1-10.0

14	Galangin	22.62	269.0455	y=3E-08x-0.1141	0.9994	0.1-10.0

Table S2. Contents of the 14 polyphenols in EAH.

NO.	Compounds	[M-H]-	Contents (mg/kg EAH)
1	Protocatechuic acid	153.0193	2.98±0.87
2	Chlorogenic acid	353.0878	1.33±0.66
3	Caffeic acid	179.0350	0.56±0.10
4	*p*-Coumaric acid	163.0401	0.76±0.09
5	Rutin	609.1461	0.90±0.06
6	Myricetin	317.0303	0.23±0.05
7	Kaempferol	285.0405	0.62±0.04
8	Luteolin	285.0405	0.38±0.09
9	Apigenin	269.0455	0.12±0.03
10	Pinocembrin	255.0663	0.29±0.05
11	3-o-acetylpinobanksin	313.0718	0.05±0.01
12	Chrysin	253.0506	0.34±0.09
13	Caffeic acid phenethyl ester	283.0976	0.22±0.02
14	Galangin	269.0455	0.53±0.04

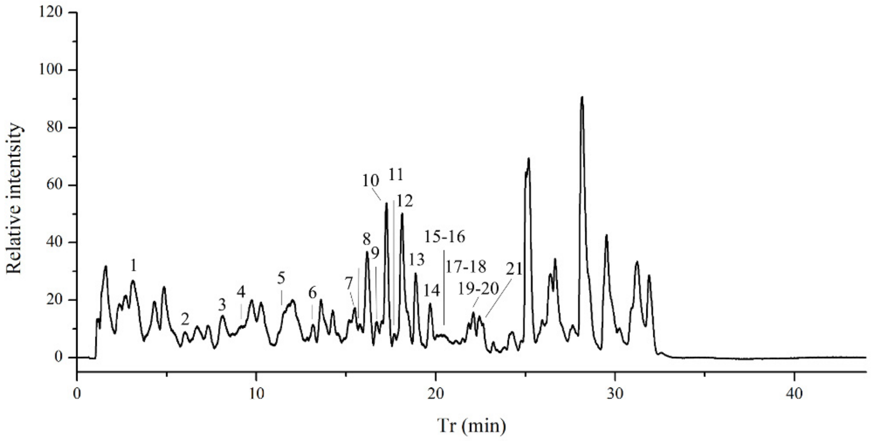

Figure S1. Total ion chromatogram in the negative ion mode of EAH

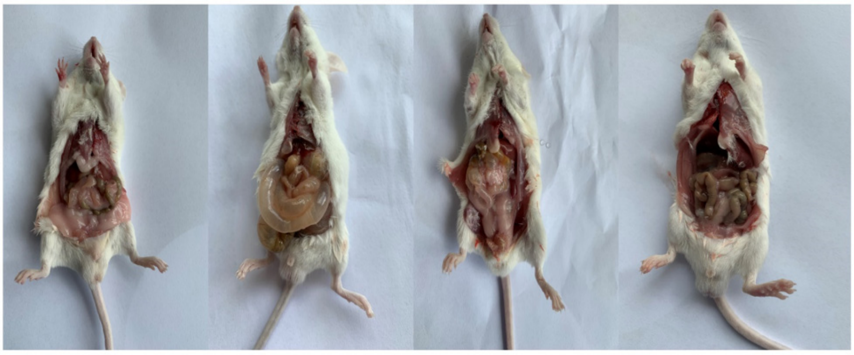

Control **Model** **Lowdose** **Highdose**

Figure S2. The flatulence of rats' gastrointestinal systems in different group

Table S3. Different species of gut microbiota of rats in different groups. (Relative abundance>2, $p<0.05$)

Treat	Taxon	Relative abundance (log10)	LDA score	P value
Control	*Bacteria.Firmicutes.Clostridia.Clostridiales.Veillonellaceae.Veillonella.Veillonella_parvula*	3.42	3.11	0.01
	Bacteria.Firmicutes.Clostridia.Clostridiales.Veillonellaceate.Veillonella	3.43	3.11	0.01
	Bacteria.Firmicutes.Bacilli.Gemellales.Gemellaceae.Gemella	2.29	2.97	0.01
	Bacteria.Firmicutes.Clostridia.Clostridiales.Veillonellaceae	3.50	3.18	0.01
	Bacteria.Firmicutes.Bacilli.Bacillales.Planococcaceae.Sporosarcina	3.91	3.56	0.02
	Bacteria.Firmicutes.Bacilli.Gemellales.Gemellaceae	2.29	2.95	0.01
Model	*Bacteria.Firmicutes.Clostridia.Clostridiales.Christensenellaceae*	3.04	2.92	0.04
LowDose	*Bacteria.Proteobacteria.Betaproteobacteria.Burkholderiales.Oxalobacteraceae*	2.23	3.56	0.00
	Bacteria.Proteobacteria.Gammaproteobacteria.Pseudomonadales	4.70	4.37	0.03
	Bacteria.Proteobacteria.Gammaproteobacteria.Pseudomonadales.Moraxellaceae.Psychrobacter	4.70	4.37	0.01
	Bacteria.Proteobacteria.Gammaproteobacteria.Pseudomonadales.Moraxellaceae	4.70	4.37	0.03
	Bacteria.Proteobacteria.Betaproteobacteria.Burkholderiales.Oxalobacteraceae.Cupriavidus	2.12	3.31	0.02
HighDose	*Bacteria.Proteobacteria.Betaproteobacteria*	4.66	4.30	0.03
	Bacteria.Firmicutes.Erysipelotrichi.Erysipelotrichales.Erysipelotrichaceae.Clostridium	4.18	3.91	0.01
	Bacteria.Firmicutes.Bacilli.Lactobacillales.Aerococcaceae.Atopostipes.Atopostipes_suicloacalis	3.54	3.22	0.02
	Bacteria.Firmicutes.Bacilli.Lactobacillales.Aerococcaceae	4.43	4.09	0.02
	Bacteria.Firmicutes.Bacilli.Lactobacillales.Aerococcaceae.Atopostipes	3.54	3.22	0.02
	Bacteria.Firmicutes.Bacilli.Bacillales.Staphylococcaceae.Macrococcus	2.54	3.14	0.04
